# Pharmacokinetic / pharmacodynamic relationships of liposomal amphotericin B and miltefosine in experimental visceral leishmaniasis

**DOI:** 10.1371/journal.pntd.0009013

**Published:** 2021-03-02

**Authors:** Andrew A. Voak, Andy Harris, Jose Miguel Coteron-Lopez, Iñigo Angulo-Barturen, Santiago Ferrer-Bazaga, Simon L. Croft, Karin Seifert

**Affiliations:** 1 Faculty of Infectious and Tropical Diseases, London School of Hygiene & Tropical Medicine, London, United Kingdom; 2 Pharmidex, London, United Kingdom; 3 Diseases of the Developing World (DDW), GlaxoSmithKline, Madrid, Spain; Institute of Postgraduate Medical Education and Research, INDIA

## Abstract

**Background:**

There is a continued need to develop effective and safe treatments for visceral leishmaniasis (VL). Preclinical studies on pharmacokinetics and pharmacodynamics of anti-infective agents, such as anti-bacterials and anti-fungals, have provided valuable information in the development and dosing of these agents. The aim of this study was to characterise the pharmacokinetic and pharmacodynamic properties of the anti-leishmanial drugs AmBisome and miltefosine in a preclinical disease model of VL.

**Methodology / Principal findings:**

BALB/c mice were infected with *L*. *donovani* (MHOM/ET/67/HU3) amastigotes. Groups of mice were treated with miltefosine (orally, multi-dose regimen) or AmBisome (intravenously, single dose regimen) or left untreated as control groups. At set time points groups of mice were killed and plasma, livers and spleens harvested. For pharmacodynamics the hepatic parasite burden was determined microscopically from tissue impression smears. For pharmacokinetics drug concentrations were measured in plasma and whole tissue homogenates by LC-MS. Unbound drug concentrations were determined by rapid equilibrium dialysis. Doses exerting maximum anti-leishmanial effects were 40 mg/kg for AmBisome and 150 mg/kg (cumulatively) for miltefosine. AmBisome displayed a wider therapeutic range than miltefosine. Dose fractionation at a total dose of 2.5 mg/kg pointed towards concentration-dependent anti-leishmanial activity of AmBisome, favouring the administration of large doses infrequently. Protein binding was >99% for miltefosine and amphotericin B in plasma and tissue homogenates.

**Conclusion / Significance:**

Using a PK/PD approach we propose optimal dosing strategies for AmBisome. Additionally, we describe pharmacokinetic and pharmacodynamic properties of miltefosine and compare our findings in a preclinical disease model to available knowledge from studies in humans. This approach also presents a strategy for improved use of animal models in the drug development process for VL.

## Author summary

Visceral leishmaniasis is a neglected tropical disease, with an estimated 200 000–400 000 cases and 20 000–40 000 deaths per year worldwide. *Leishmania* parasites proliferate in spleen, liver and bone marrow, and the disease is usually fatal if untreated. Despite progress in the development of new therapies in recent years there is still a need to develop effective and safe treatments. Characterising pharmacokinetic and pharmacodynamic properties and their relationships has provided valuable information in the development and dosing of anti-infective agents, such as anti-bacterials and anti-fungals. The present study characterised these properties of the anti-leishmanial drugs AmBisome and miltefosine in a mouse model of visceral leishmaniasis. We propose optimal dosing strategies for AmBisome and present a new approach for investigating anti-leishmanial drug action, which can be applied to the development of new treatments for visceral leishmaniasis.

## Introduction

Visceral leishmaniasis (VL) is a vector-borne neglected tropical disease (NTD) caused by protozoan parasites of the genus *Leishmania*. The disease, which is fatal if untreated, presents with symptoms and signs of persistent systemic infection, such as fatigue and weight loss, and enlarged nymph nodes, spleen and liver, due to parasitic invasion of the mononuclear phagocyte system [1]. Recent estimates suggest that there are 200 000–400 000 cases and 20 000–40 000 deaths per year worldwide. Over 90% of cases occur in India, Bangladesh, Sudan, South Sudan, Brazil and Ethiopia [2]. Drug toxicity, challenging routes of drug administration, drug stability in hot climates, and geographical differences in clinical response to treatment remain a challenge to satisfactory VL therapy [3,4]. Development of safe and effective new drugs for VL necessitates the use of relevant approaches during lead optimisation. Pharmacokinetics and pharmacodynamics (PK/PD) provides a conceptual framework for improving knowledge of the biological basis of PD effects, which can aid in the development of new drugs and improved use of existing ones [5].

PK/PD concepts were initially identified for anti-bacterial agents, where different measures of exposure have been linked to the anti-microbial activity of different drug classes. These different PK/PD indices (time above minimum inhibitory concentration [T>MIC], maximum concentration to MIC ratio [C_max_/MIC], area under the curve to MIC ratio [AUC/MIC]) indicate either time-dependent or concentration-dependent drug action and translate into different dosing regimens [6–9].

Preclinical PK/PD studies have provided valuable information in the development of anti-infective agents [10], but available knowledge for anti-leishmanial drugs is limited [11]. Here we investigated maximally effective dosing regimens and dose-response effects, PK and tissue distribution, dose fractionation, and protein binding after administration of AmBisome and miltefosine in a preclinical disease model of VL. Miltefosine, an alkylphosphocholine, and AmBisome, a unilamellar liposomal formulation of amphotericin B, are two clinically used anti-leishmanial drugs [4,12,13]. These were chosen to enable the development of a novel approach for anti-leishmanial drug development and comparison of findings to available knowledge from studies in humans.

## Methods

### Ethics statement

Experiments involving animals were carried out under license in accordance with the Animals (Scientific Procedures) Act of 1986 (UK Home Office Project license PPL70/8207) following approval by the Animal Welfare and Ethics Review Board at LSHTM.

### Drugs and reagents

AmBisome was purchased from Gilead (Cambridge, UK). The powder was reconstituted in sterile water following the manufacturer’s directions and further dilutions prepared in 5% glucose. Miltefosine was obtained from Paladin Labs Inc. (Montreal, Canada). Amphotericin B (Vetranal analytical standard), tolbutamide, dimethyl sulfoxide (DMSO), sodium dodecyl sulfate (SDS) and acetonitrile were obtained from Sigma (UK). Heparin was obtained from John Bell & Croydon (UK). Methanol (high-performance liquid chromatography [HPLC] grade), 0.1% formic acid in water (liquid chromatograph-mass spectrometry [LC-MS] grade) and water (LC-MS grade) were purchased from Fisher Scientific UK, Ltd. (UK).

### In vivo experiments–infection, randomisation and endpoints

Female BALB/c (Charles River, UK) and Rag 1 (B6) knockout mice (LSHTM breeding colony) were maintained under specific-pathogen-free conditions in individually ventilated cages and exposed to 12-h-light-12-h-dark cycles. Standard rodent diet (RM no. 1 expanded) and filtered tap water were supplied *ad libitum*. Parasites were maintained in Rag-1 (B6) KO mice and amastigotes harvested from spleens >40 days after infection. Mice (6 to 10 weeks of age at the start of experiments) were infected by intravenous (i.v.) injection of 2 x 10^7^ parasites (*L*. *donovani* MHOM/ET/67/HU3) as described previously [14]. Treatment of BALB/c mice started 10 days (miltefosine) or 14 days (AmBisome) after infection, at doses and dosing regimens indicated below. Timepoints for treatment start were chosen to ensure that drug effects were evaluated within the time window, in which parasites replicate in the liver and pathology is established. Prior to the administration of drugs mice were weighed and randomized into the different treatment groups, using a random number generator. The average weight of mice in each experiment was used for dose calculations. Untreated groups of mice were included as controls. At experimental endpoints mice were weighed and humanely killed by exsanguination under terminal anaesthesia. Blood was collected by cardiac puncture in Eppendorf tubes containing heparin, and plasma was harvested by centrifugation. Livers and spleens were removed and their weight recorded. Plasma and tissue samples were stored at -80°C until further processing. Parasite burden was determined by microscopy in 100% methanol-fixed tissue impression smears stained with 10% Giemsa. Parasite burden was expressed in Leishman-Donovan units (LDU), calculated by the formula no. of parasites per host cell nucleus x organ weight in mg [15].

### In vivo experiments–dosing regimens and data analysis

Miltefosine was administered orally (p.o.) by gavage of a 0.2 mL bolus at a dose of 30 mg/kg in repeated daily dose schedules to evaluate the efficacy of different dosing frequencies and as single dose to determine concentration-time profiles. Dose-response evaluation included daily doses of 30 mg/kg, 15 mg/kg, 7.5 mg/kg, 3.8 mg/kg and 1.9 mg/kg over 5 days. To determine the maximum effective dose AmBisome was administered intravenously (i.v.) in a 0.2 mL bolus injection into a tail vein at single doses of 5 mg/kg, 10 mg/kg, 20 mg/kg and 40 mg/kg and to evaluate concentration-time profiles at a single dose of 40 mg/kg. To determine which PK parameter is driving anti-leishmanial efficacy we administered total doses of 1.25 mg/kg and 2.5 mg/kg, either as a single injection or split into 2 (q24 hours) or 4 (q12 hours) injections.

Percentage inhibition of parasite burden in drug-treated groups was calculated in relation to an untreated control group. Additionally, data was analysed and plotted based on the absolute parasite burden in log10 LDU.

### Processing of samples for drug quantification

Tissue and plasma samples were thawed at room temperature. Tissue samples were homogenised in a Bullet blender (Next Advance, UK) as described previously [14]. Samples containing amphotericin B were processed as described previously [14] using the internal standard (IS) as described below for miltefosine. For analysis of miltefosine blanks (control matrix samples plus IS), calibration standards, quality control (QC) samples and study samples, 50 μL of tissue homogenate or plasma was diluted with 250 μL of IS solution (2000 ng/mL tolbutamide in acetonitrile). After shaking at room temperature for 10 minutes at 200 rpm, dilutions were centrifuged at 4 150 x g for 15 minutes at 4°C. Supernatants were transferred to 96-well plates and stored at -80°C. Dilution of study samples, when necessary, was carried out as described previously [14].

### Preparation of calibration standards and QC samples

Calibration standards and QC samples for amphotericin B containing samples were prepared as described previously [14]. For miltefosine a stock solution (1 mg/mL) was prepared in a mixture of 1:1 (v/v) methanol:water. From that standard spiking solutions were prepared by serial dilution in methanol:water (1:1, v/v). Calibration standards were prepared at 12 concentrations by mixing 5 μL of the spiking solutions with 45 μL of blank tissue homogenate or plasma, with the matrix matching that of the study samples to be analysed. QC samples at selected concentrations were prepared in replicates in similar fashion. All samples were processed as described above.

### LC-MS analytical conditions

Samples containing amphotericin B were analysed as described previously [14]. Total amphotericin B levels were measured throughout the experiments. All samples were analysed for miltefosine using an Agilent 1200 HPLC combined with an Agilent 6410A triple quadrupole mass spectrometer (both Agilent, UK). A mobile phase of water / 0.1% formic acid (channel A) and methanol / 0.1% formic acid (channel B) at 0.6 mL/min was used to elute sample components from a Luna column packed with 3 μm C_8_ material (2.1mm x 50mm @50°C; Phenomenex, UK). The mobile phase composition was initially 2% B, programmed to increase linearly to 60% B at 0.60 min. after injection and then linearly to 95% B at 2.1min. The composition was maintained at 95% B for a further 0.4 min. before returning to its initial 2% B at 2.60 min. post-injection. Miltefosine was detected monitoring the transition m/z 408.3 ➔ m/z 124.8, with internal standard tolbutamide detected monitoring m/z 271.1 ➔ m/z 91.

Analyte concentrations were quantified against calibration standards prepared in matched control matrix, with aliquots of sample and standard being injected typically in the range 1–5 μL, depending on expected study sample concentration. Calibration curves were constructed by using fits and weighting to minimise residuals for back-calculated concentrations for standards over the range of concentrations encountered in the study samples. Calibration standards with residuals >20% were excluded from the curve fitting. Calibration curves consisted of standards with between 7 and 11 different concentrations. As well as calibration curve quality, analytical batch acceptance was based upon the results of analyses of QC samples. Data obtained for QC samples is summarized in S1 Table.

### Statistical analysis

Statistical significance between groups was analysed by one-way analysis of variance (ANOVA) assuming a Gaussian distribution, followed by Sidak’s multiple-comparison test for selected groups (GraphPad Prism 6). A *P* value of ≤ 0.05 was considered statistically significant.

### Pharmacokinetic analysis

Noncompartmental analysis (NCA) was performed with Phoenix Win Nonlin v6.3 (Certara, United Kingdom).

### Rapid equilibrium dialysis

Plasma, livers and spleens were harvested from mice 24 hours after the administration of a single dose of 40 mg/kg AmBisome or within 1 hour after the first and last dose of a total of 5 daily doses of 30 mg/kg miltefosine. Tissue homogenates were prepared as described above. Percentages of unbound drug concentrations were measured by rapid equilibrium dialysis (RED) using single use plates with inserts (Fisher Scientific, UK) according to the manufacturer’s instructions. Briefly, plasma or tissue homogenate was plated in one half of a sample chamber insert, with dialysis buffer loaded into the other half of the insert. The plates were sealed and incubated for 4 hrs at 37°C with agitation. Following incubation, the sample chamber contents were removed and mixed with an equal volume of dialysis buffer, and likewise the buffer chamber contents were mixed with plasma/homogenate from untreated mice. Sample and buffer fractions were then protein precipitated, the drug extracted, and samples analysed using LC-MS as described above. Extractions were compared to standard curves and QCs generated as described above.

## Results

### Pharmacodynamics and biodistribution of repeated dose miltefosine and single dose AmBisome

**Fig 1:**
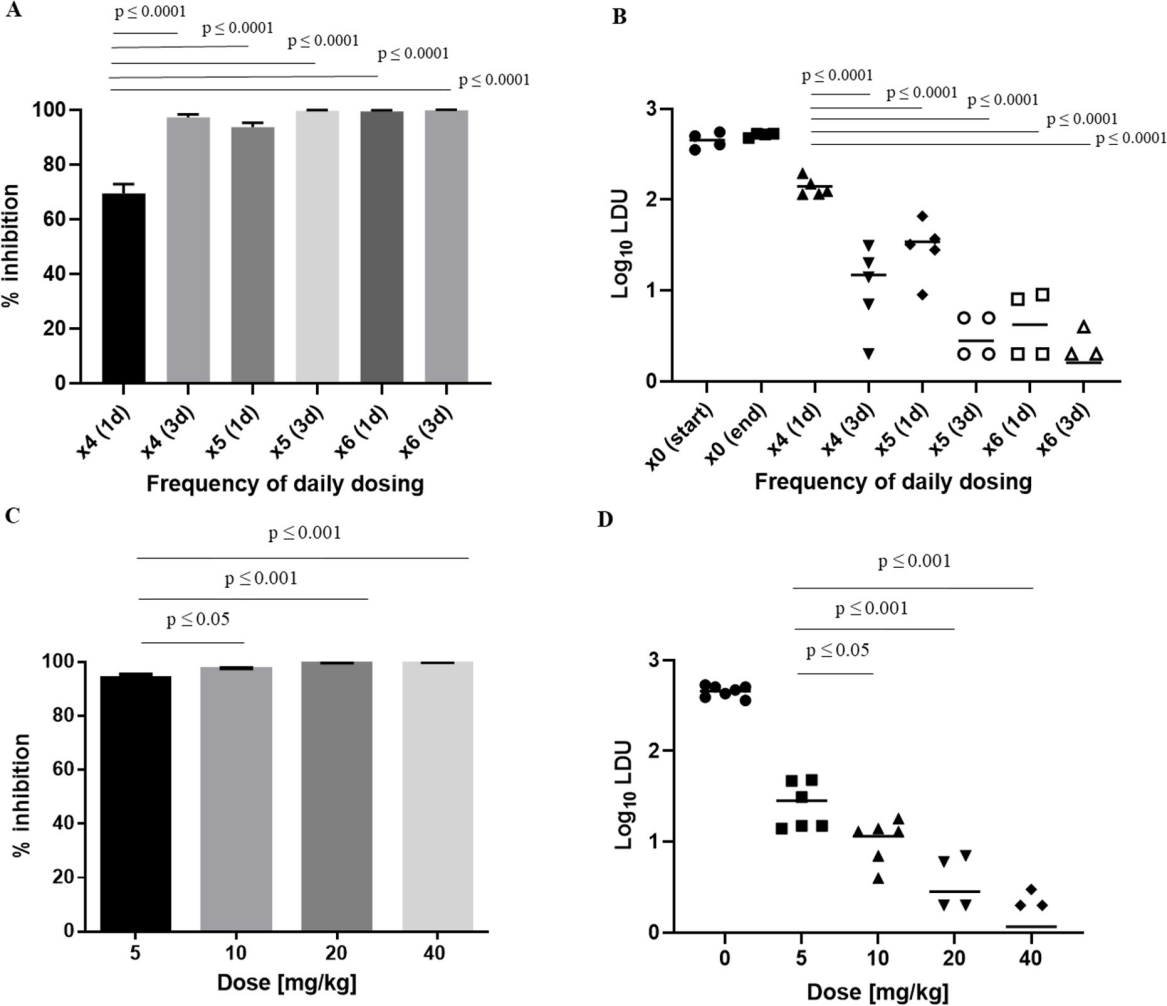
Dose-response data for repeated dose miltefosine and single dose AmBisome. Groups of BALB/c mice were treated with 30 mg/kg miltefosine at different dosing frequencies (n = 5; A, B) or different single doses of AmBisome (n = 6; C, D). Treatment outcome is expressed as percentage inhibition, where columns represent group means and error bars standard deviations (A, C), or parasite burden in log10 LDU, where horizontal lines represent group means and symbols data from individual mice (B, D). Following miltefosine treatment parasite burden was evaluated either 1 day (1d) or 3 days (3d) after the last dose of 4 (x4), 5 (x5) or 6 (x6) doses. Mean LDUs +/- standard deviation in untreated control groups were 454 +/- 91 and 515 +/- 26 at corresponding time points to 4 and 6 doses for data shown in (A), and 456 +/- 65 for data shown in (C). Log10 LDUs in untreated control groups corresponding to time points of 4 and 6 doses in (B) are denoted as x0 (start) and x0 (end).

For miltefosine we first investigated drug efficacy after different dosing frequencies of once daily dosing for either 4, 5 or 6 days, and determined the parasite burden 1 or 3 days after the last dose. The percentage inhibition of parasite burden in LDUs was 69.1 ± 7.5%, 93.8 ± 1.7% and 99.2 ± 0.3% on day 1 after the last dose and 97.3 ± 1.0%, 99.5 ± 0.2% and 99.7 ± 0.1% on day 3 after the last dose (Fig 1A). The corresponding absolute parasite burden in log10 LDU was 2.1 ± 0.1, 1.5 ± 0.3 and 0.5 ± 0.4 on day 1 after the last dose and 1.0 ± 0.5, 0.4 ± 0.3 and 0.2 ± 0.3 on day 3 after the last dose. Amastigotes were detected in livers from all animals of groups with ≥ 1.0 mean log10 LDU, whereas no amastigotes were detected in livers from some animals in groups with parasite burdens of ≤ 0.5 log 10 LDUs (Fig 1B).

**Fig 2:**
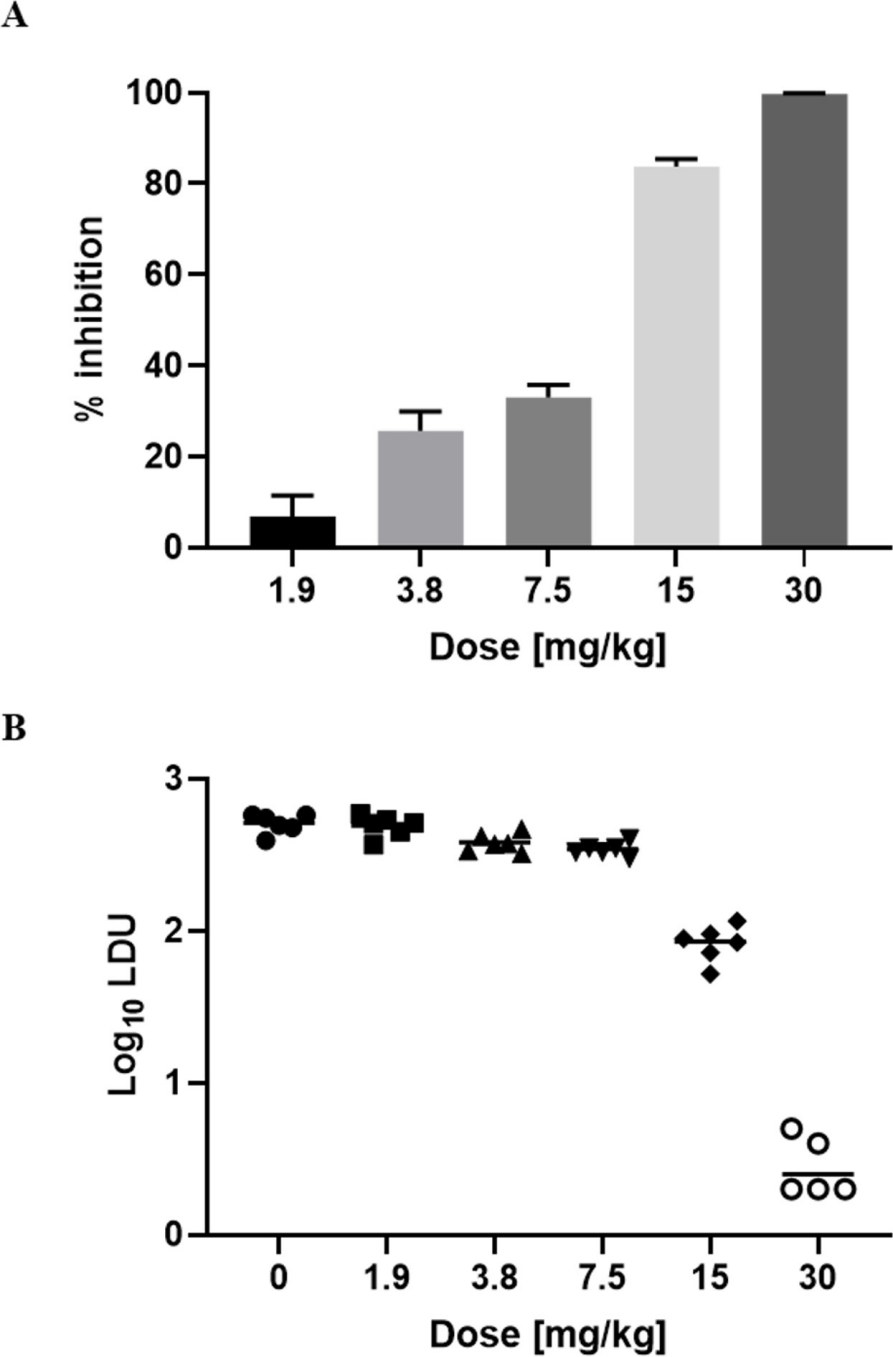
Dose-response data for miltefosine. Groups of BALB/c mice were treated over 5 days at daily doses shown in the graphs. Treatment outcome is expressed as percentage inhibition, where columns represent group means (n = 6) and error bars standard deviations (A), or parasite burden in log10 LDU, where horizontal lines represent group means and symbols data from individual mice (B). Mean LDU +/- standard deviation in the untreated control group in (A) were 512 +/- 72.

Next, we evaluated the dose-response effect of miltefosine, employing the most effective dosing frequency, in parallel with plasma sampling at defined time points after the first and last dose. Percentage inhibition >90% of parasite burden in LDUs was only achieved at the highest dose of 30 mg/kg (Fig 2A). This was also the only dose at which the parasite burden in log10 LDU was < 1.0 (Fig 2B) and at which no amastigotes were detected in the liver of one of the mice. Plasma concentrations indicated drug accumulation over time (Table 1).

**Table 1:** Drug concentrations in plasma following administration of miltefosine.

	Miltefosine concentration in plasma [μg/mL], mean +/- SD at doses of:							
	30 mg/kg		15 mg/kg		7.5 mg/kg		3.8 mg/kg	
Hour	x1	x5	x1	x5	x1	x5	x1	x5
**1**	7.5 +/- 0.7	35.8 +/- 1.3	3.2 +/- 0.7	*19.5 +/- 2.2	2.0 +/- 0.1	10.0 +/- 0.5	*0.9 +/- 0.0	*5.3 +/- 0.3
**8**	11.8 +/- 1.2	*44.9 +/- 2.2	6.6 +/- 0.5	27.6 +/- 4.7	3.6 +/- 0.2	11.2 +/- 1.1	1.7 +/- 0.0	5.1 +/- 0.6
**24**	12.1 +/- 0.4	36.8 +/- 5.1	6.1 +/- 0.7	17.1 +/- 3.3	2.8 +/- 0.3	8.1 +/- 1.0	1.4 +/- 0.0	*3.9 +/- 0.9

Plasma was sampled at indicated timepoints (hours) after administration of the first dose (x1) and last dose (x5) of multiple dose levels. A composite sampling design was applied, where samples 1 and 24 hours after drug administration were taken from the same 3 animals of a group of 6 in total and samples 8 hours after drug administration from the other 3 animals.

*indicates that data is derived from two samples only.

For AmBisome we determined the maximum effective dose by administering single doses of up to 40 mg/kg and evaluated the hepatic parasite burden 2 days later. The chosen regimen was based on the demonstration of maximum kill 2 days after administration of a single dose of 10 mg/kg AmBisome [14] and a dose-response study with doses ranging from 0.625 mg/kg to 10 mg/kg (S3 Table). The percentage inhibition of parasite burden at the different doses was 93.8 ± 1.5%, 97.4 ± 0.5%, 99.4 ± 0.3% and 99.7 ± 0.1% (Fig 1C). The corresponding parasite burden based on log10 LDU was 1.4 ± 0.3, 1.0 ± 0.2, 0.4 ± 0.4 and 0.2 ± 0.2, respectively (Fig 1D). Additionally, at doses of 20 mg/kg and 40 mg/kg no amastigotes were detected in livers of 2/6 and 3/6 animals.

**Fig 3:**
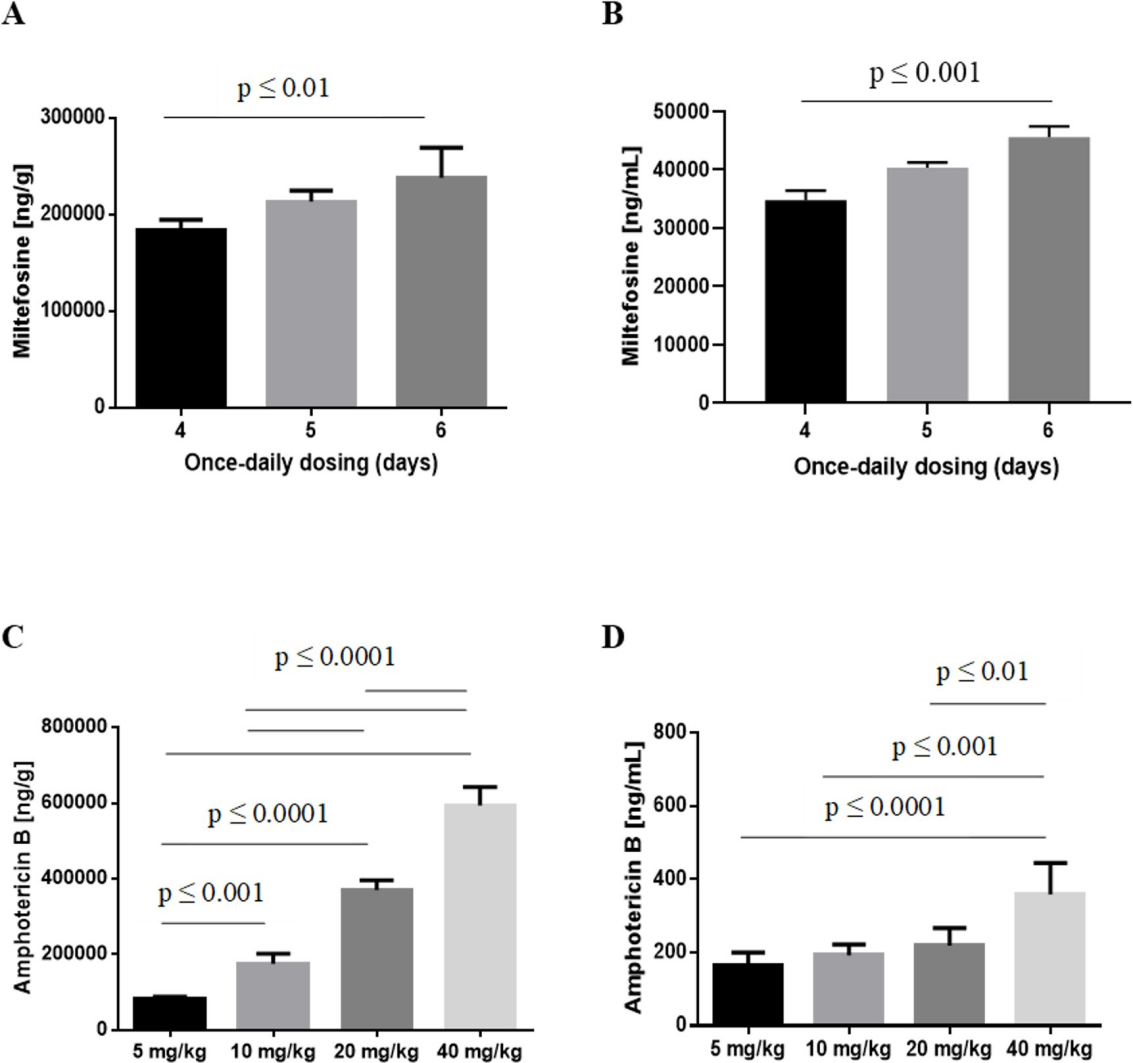
Drug concentrations in plasma and liver following administration of repeated dose miltefosine and single dose AmBisome. Drug concentrations in livers (A, C) and plasma (B, D) were measured in the same animals for which dose response data is given in Fig 1. Columns represent group means and error bars standard deviations, following treatment with 30 mg/kg miltefosine (n = 5; A, B) or different doses of AmBisome, as indicated (n = 6; C, D).

In parallel to determining drug efficacy we measured concentrations of miltefosine and amphotericin B in livers and plasma from the same mice, in which treatment outcome was determined. This data is shown in Fig 3. Tabulated results for both parasite burden and drug concentrations are provided in S2 Table for miltefosine and S3 Table for amphotericin B.

### Pharmacokinetics and protein binding of amphotericin B and miltefosine

**Fig 4:**
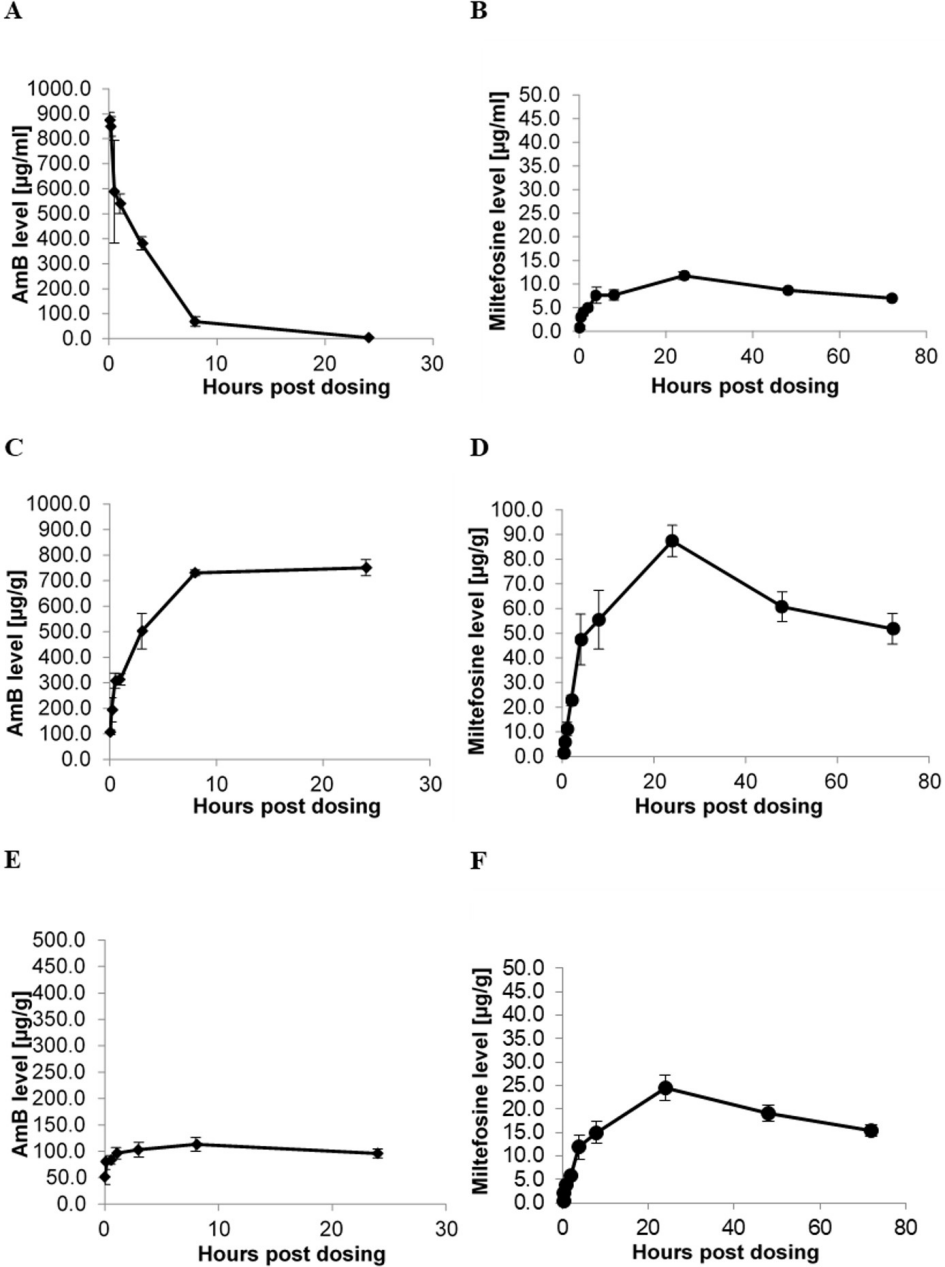
Concentration-time profiles after single doses of AmBisome and miltefosine. Single doses of 40 mg/kg AmBisome (A, C, E) and 30 mg/kg miltefosine (B, D, F) were administered and drug concentrations determined in plasma (A, B), liver (C, D) and spleen (E, F) at 0.05, 0.17, 0.5, 1, 3, 8 and 24 hours (A, C, E) or at 0.25, 0.5, 1, 2, 4, 8, 24, 48 and 72 hours post dose (B, D, F). Each data point represents the group mean +/- standard deviation (n = 3). Mean LDU and log10 LDU +/- standard deviation in untreated control groups were 791 +/- 35 and 2.9 +/- 0.0 for data shown in A, C and E, and 367 +/- 27 and 2.6 +/- 0.0 for data shown in B, D, and F.

The shape of the concentration-time profiles in plasma and organs (liver, spleen) was discordant for amphotericin B, whereas miltefosine displayed similar profiles in plasma, liver and spleen (Fig 4). Plasma half-life and AUC_last_ were 63.71 hours and 640.44 hours*μg/mL for miltefosine and 3.24 hours and 2 853.73 hours*μg/mL for amphotericin B. PK parameters and tabulated results are provided in Table 2 and S4 Table, respectively.

**Table 2:** PK parameters of single dose miltefosine and AmBisome.

	Miltefosine (30 mg/kg p.o.)			AmBisome (40 mg/kg i.v.)		
Parameter	Plasma	Liver	Spleen	Plasma	Liver	Spleen
t_1/2,_ h	63.71	63.60	71.66	3.24	ND	ND
T_max,_ hr	24.00	24.00	24.00	0.05	24.00	8.00
C_max,_ μg/mL	11.80	87.40	24.50	874.90	750.80	113.20
AUC_last,_ hr*μg/mL	640.44	4568.79	1331.24	2853.73	16010.44	2492.80

For both drugs the percentage of bound drug was > 99% in all matrices (Table 3).

**Table 3:** Protein binding of amphotericin B and miltefosine in plasma and tissue.

			**% bound (mean +/- SD, n = 3)**		
	**Dosing**	**Hours post dose**	**Plasma**	**Liver**	**Spleen**
AmBisome	40 mg/kg x1	24	99.60 +/- 0.41	99.98 +/- 0.00	99.96 +/- 0.02
Miltefosine	30 mg/kg x1	1	99.92 +/- 0.06	99.97 +/- 0.00	99.93 +/- 0.07
Miltefosine	30 mg/kg x5	1	99.98 +/- 0.00	99.99 +/- 0.00	99.99 +/- 0.00

### Dose fractionation of AmBisome

**Fig 5:**
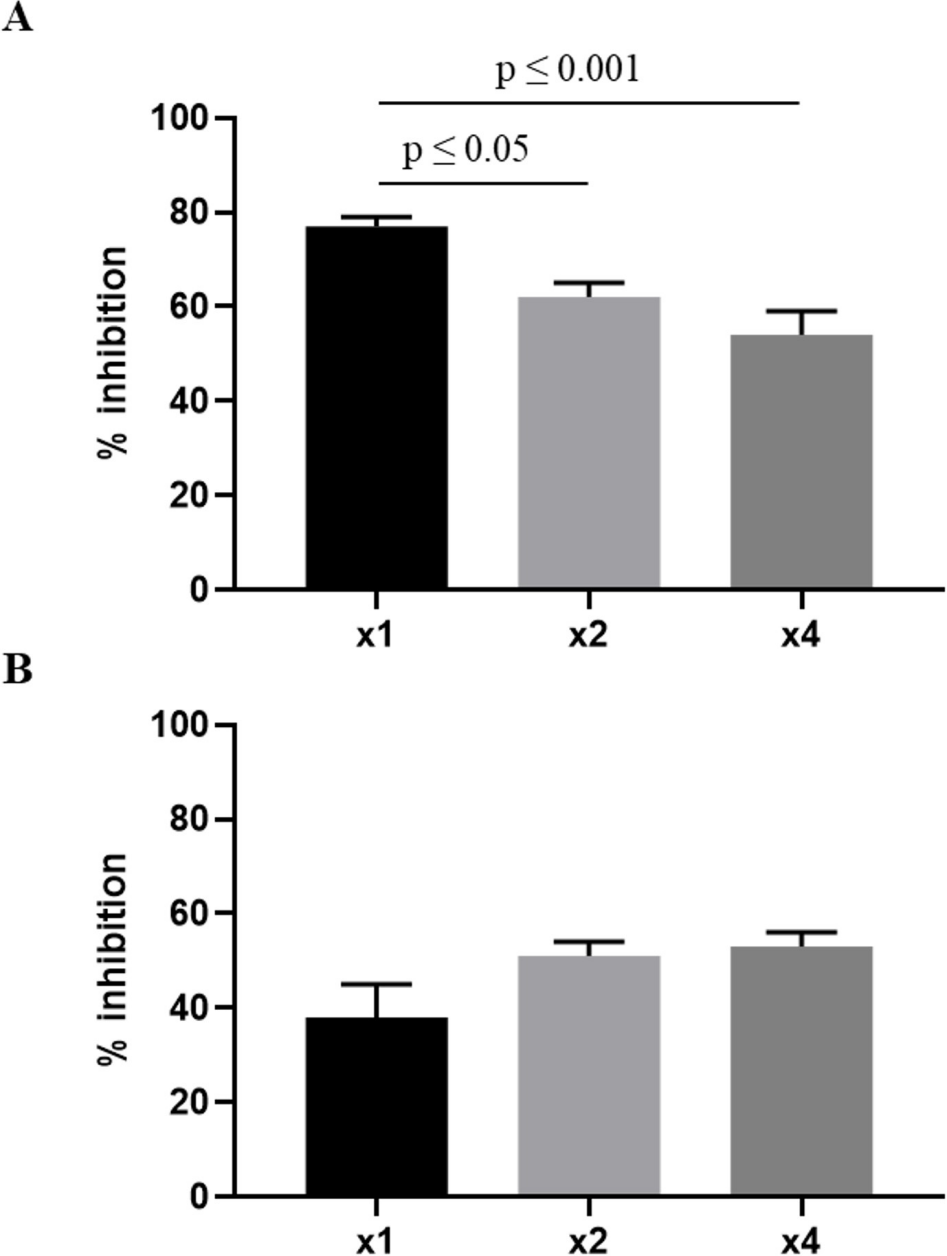
Dose fractionation of AmBisome. Total doses of 2.5 mg/kg (A) and 1.25 mg/kg (B) were administered either as one dose (x1), one-half of the dose every 24 hours (x2) or one-fourth of the dose every 12 hours (x4). Treatment outcome is expressed as percentage inhibition, where columns represent group means (n = 6) and error bars standard deviations. Mean LDU +/- standard deviation in an untreated control group was 655 +/- 76. Data for the 2.5 mg/kg dose is representative of 2 separate experiments.

To determine which PK parameter is driving anti-leishmanial efficacy we carried out dose fractionation at doses, which correspond to the ED_50_ and ED_70_ values estimated from the initial dose-response study in S3 Table. The parasite burden in the liver was significantly lower when the total dose of 2.5 mg/kg was administered once compared to administrations as one-half of the dose every 24 hours or as one-fourth of the dose every 12 hours. However, no significant difference between the different dosing regimens was observed at the lower total dose of 1.25 mg/kg (Fig 5). Overall, this data points towards a concentration-dependent (AUC driven) pharmacodynamic effect.

## Discussion

A main purpose of pre-clinical PK/PD studies in drug development is to identify optimal dosing schedules [10]. Here we have used an established experimental model to characterise PK/PD relationships of two clinically used anti-leishmanial drugs. Infection of BALB/c mice with the *L*. *donovani* strain used here leads to different kinetics and magnitudes of the parasite burden in liver and spleen [16–18]. Specifically, in the liver a rapid increase in parasite burden is observed during the first 3 weeks after infection, followed by clearance of parasites. In the spleen, which remains chronically infected, the parasite burden only increases after the first 2 weeks and peaks around 4 weeks after infection. This necessitates the use of different time windows to evaluate a compound’s anti-leishmanial effect against a multiplying parasite population in these two organs. We focussed our pharmacodynamic investigations on the hepatic parasite burden, which is most often used to measure treatment efficacy during preclinical anti-leishmanial drug development [19]. Some investigations of the drugs’ tissue concentrations were additionally carried out in the spleen to maximise knowledge gained from experiments involving animals.

It is standard practice to estimate efficacy of anti-leishmanial compounds in BALB/c mice by determining the parasite burden in LDU [15] and calculating the percentage reduction of parasite load in drug-treated animals in relation to a control group [19]. Here we additionally used an alternative analysis based on the (absolute) parasite burden in log10 LDU. Based on this analysis single and cumulative doses of AmBisome and miltefosine exerting maximum anti-parasitic effects were 40 mg/kg and 150 mg/kg, respectively.

Dose-response data revealed a dosing window ranging from 5 to 40 mg/kg (8-fold range) in which AmBisome inhibited the parasite burden by over 90%, which is the minimal target profile for new chemical entities required by the Drugs for Neglected Diseases Initiative (DNDi) according to the target product profile for VL. Using DNDi’s optimal target profile of >95% reduction [19] as cut-off still leaves a 4-fold dosing window ranging from 10 to 40 mg/kg, with a reduction of 94% at 5 mg/kg. In contrast, miltefosine displayed an inhibition of over 90% and 95% only after cumulative doses of 120, 150 and 180 mg/kg. Higher doses were not administered to minimise the risk of overt toxic effects, which are known to occur at higher doses ([20] and personal observations). The dosing window in which a parasite burden of log10 LDU <1 was achieved with AmBisome ranged from 20 to 40 mg/kg (2-fold range), whereas for miltefosine this was only observed at cumulative doses of 150 mg/kg and 180 mg/kg. This data demonstrates a wider therapeutic range of AmBisome compared to miltefosine.

Drug distribution to infected sites is an important consideration in anti-infective PK/PD [21,22]. In VL *Leishmania* parasites reside and multiply within parasitophorous vacuoles (PV) of macrophages in bone marrow, liver and spleen [23]. Hence, drug concentrations closest to the site of action are those at subcellular (PV) level. However, measuring drug concentrations at cellular and subcellular level *in vivo* is technically challenging. We therefore determined drug concentrations in tissue homogenates, acknowledging that these are hybrid concentrations of drug in different tissue compartments [6,22]. Nonetheless they are of value in determining the overall distribution of a drug [24] and we used them to compare the time course of drug distribution between plasma, liver and spleen. In line with previous reports in anti-fungal therapy [21] and in agreement with previous data from our lab [14] hysteresis, *i*.*e*. discordance in the shape of the amphotericin B-concentration-time profiles between plasma and tissue, was observed following administration of AmBisome. This pattern can be explained by clearance of amphotericin B-containing liposomes from the bloodstream by the reticuloendothelial system (RES) and distribution to tissues [14,25]. In contrast similar shapes of the concentration-time curves in plasma and tissue were observed for miltefosine. Preferential distribution to liver and spleen of amphotericin B has also been reported in autopsy material from patients, who were treated with liposomal amphotericin B for fungal infections within the last 72 hours of their life [26], but direct comparison of drug levels between this data in humans and our study are complicated by use of different doses, dosing regimens and above described hysteresis.

Dose fractionation studies are a powerful tool to examine the driver of drug efficacy [27]. Using this approach amphotericin B’s anti-fungal action was shown to be C_max_ driven [28] and concentration-dependent fungicidal pharmacodynamics was observed in preclinical studies [13]. Our data points towards a concentration-dependent (AUC driven) pharmacodynamic effect, which supports dosing strategies associated with the administration of large doses infrequently [28], whereby choice of dose and dosing regimens in humans needs to be balanced against tolerability and rate of adverse drug reactions. The potential value of intermittent administration of liposomal amphotericin B, as short course or single dose, has also been pointed out based on the formulation’s prolonged mean residence time in tissues [29]. In human VL non-inferiority of single-dose liposomal amphotericin B to conventional therapy was demonstrated in a randomised clinical trial on the Indian subcontinent [30] where a single dose of liposomal amphotericin B is now considered highly effective [12]. A multi-centre randomised trial in Eastern Africa reported higher parasite clearance rates from peripheral blood when patients were treated with multiple doses of AmBisome compared to a single dose [31]. However, the study was terminated prematurely due to an unexpected low efficacy of all treatment regimens and pharmacodynamic conclusions were based on a small number of patients. Additionally, measuring parasite load circulating in blood is an indirect method, whereas the subject of our study was the parasite burden in a target tissue. Dose fractionation studies with miltefosine were hampered by its long half-life and accumulation in plasma. Hence it was not possible to formally define the PK/PD driver for miltefosine. Clinical studies in VL indicated that the mechanism of action of miltefosine is defined by a time-dependent killing effect [32,33], which was further supported by the observation that duration of miltefosine treatment was found to be important in the early dose-finding studies [34].

Determination of protein binding is an integral part of pharmacological studies as protein binding impacts on distribution and elimination processes. It is also generally accepted that free (unbound) drug concentrations at the site of action are responsible for the PD effect of anti-infective agents [22,35,36]. Due to the technical difficulties of determining free drug concentrations at cellular and subcellular levels *in vivo* we measured protein binding in plasma and tissue homogenates from *L*. *donovani* infected and treated BALB/c mice. Using rapid equilibrium dialysis [37] we observed protein binding of >99% for amphotericin B and miltefosine. In human plasma protein binding of >99% and 96–98% was observed for amphotericin B [25] and miltefosine [38], respectively.

Finally, we compared plasma concentrations of miltefosine measured here to previously reported *in vitro* drug susceptibilities of intracellular amastigotes of the same parasite strain [39,40]. Total plasma concentrations of 35–46 μg/mL after cumulative doses of 120, 150 and 180 mg/kg miltefosine clearly exceeded the reported EC_90_ values of 5.8–9.1 μg/mL. Total plasma concentrations after a cumulative dose of 75 mg/kg also exceeded the *in vitro* EC_90_ values, but only provided a partial treatment response. However, due to the intracellular location of *Leishmania* parasites intracellular drug accumulation and drug transport mechanisms at cellular and subcellular level play a pivotal role in drug action and target site concentrations may differ markedly from those measured in plasma [21,34].

In summary, we have applied PK/PD approaches to investigate anti-leishmanial drug action and to support recommendations for dosing regimens. Concentration-dependent drug action of AmBisome fits well with established knowledge about the anti-infective properties of amphotericin B and can be exploited in combination drug regimens. If similar patterns are seen in a chronic model of the disease remains subject of future studies.

## Supporting information

S1 Table Summary data for QC samples. Data is summarized for all QC samples across different experiments to illustrate method accuracy.
(DOCX)

S2 Table Parasite burden and drug concentrations in tissue and plasma after administration of repeated doses of miltefosine. Data from experiment 2 presented here corresponds to data graphically presented in Figs 1 and 3 in the main manuscript. Data provided here in LDU was used along with the parasite burden in untreated control groups to calculate the % inhibition shown in Fig 1A. Additional data are shown here for an experiment comparing the hepatic parasite burden after 3 and 5 daily doses of miltefosine, and for drug concentrations in the spleen compared to plasma and liver. * and ** denote that in 1 and 2 animals of the respective groups no amastigotes were detected in the liver.
(DOCX)

S3 Table Parasite burden and drug concentrations in tissue and plasma after administration of single dose AmBisome. Data from experiment 2 presented here corresponds to data graphically presented in Figs 1 and 3 in the main manuscript. Data provided here in LDU was used along with the parasite burden in untreated control groups to calculate the % inhibition shown in Fig 1C. Additional data are shown for an initial dose response experiment which included a lower dose range, and for drug concentrations in the spleen compared to plasma and liver. * and ** denote that no amastigotes were detected in 2 and 3 animals of the respective groups.
(DOCX)

S4 Table Drug-concentration-time profiles after administration of single doses of AmBisome and miltefosine. Data presented here corresponds to data graphically presented in Fig 4.
(DOCX)
