# Supplementary material for: Pharmacokinetic / pharmacodynamic relationships of liposomal amphotericin B and miltefosine in experimental visceral leishmaniasis
Source: PLoS Negl Trop Dis. 2021 Mar 2;15(3):e0009013. doi: 10.1371/journal.pntd.0009013 (PMC7924795; doi:10.1371/journal.pntd.0009013)
Supplement: S1 Table — Data is summarized for all QC samples across different experiments to illustrate method accuracy. (DOCX) [file pntd.0009013.s001.docx]

**S1 Table**

| Compound | Matrix | Number QCs | QC accuracy (relative to nominal) | | | |
| --- | --- | --- | --- | --- | --- | --- |
|  |  |  | within +/- 30% | within +/- 25% | within +/- 15% | within +/- 10% |
| Amphotericin | Plasma | 14 | 13 | 13 | 12 | 9 |
|  | Liver | 25 | 21 | 18 | 14 | 11 |
|  | Spleen | 30 | 30 | 29 | 23 | 18 |
| Miltefosine | Plasma | 10 | 10 | 9 | 6 | 2 |
|  | Liver | 25 | 25 | 25 | 25 | 18 |
|  | Spleen | 20 | 20 | 20 | 19 | 12 |
