# Supplementary material for: Pharmacokinetic / pharmacodynamic relationships of liposomal amphotericin B and miltefosine in experimental visceral leishmaniasis
Source: PLoS Negl Trop Dis. 2021 Mar 2;15(3):e0009013. doi: 10.1371/journal.pntd.0009013 (PMC7924795; doi:10.1371/journal.pntd.0009013)
Supplement: S2 Table — Data from experiment 2 presented here corresponds to data graphically presented in Figs 1 and 3 in the main manuscript. Data provided here in LDU was used along with the parasite burden in untreated control groups to calculate the % inhibition shown in Fig 1A. Additional data are shown here for an experiment comparing the hepatic parasite burden after 3 and 5 daily doses of miltefosine, and for drug concentrations in the spleen compared to plasma and liver. * and ** denote that in 1 and 2 animals of the respective groups no amastigotes were detected in the liver. (DOCX) [file pntd.0009013.s002.docx]

**S2 Table**

| **Expt.** | **Sacrifice (after last dose)** |  | **Number of daily miltefosine doses at 30 mg/kg** | | | |
| --- | --- | --- | --- | --- | --- | --- |
|  |  |  | **x 3** | **x 4** | **x 5** | **x 6** |
|  |  |  | **Parasite burden, mean +/- SD** | | | |
| **1** | **1 day** | **LDU** | 526 +/- 13 | ND | 161 +/- 41 | ND |
|  | **3 days** | **LDU** | 249 +/- 53 | ND | 28 +/- 16 | ND |
|  | **1 day** | **Log10 LDU** | 2.7 +/- 0.0 | ND | 2.2 +/- 0.1 | ND |
|  | **3 days** | **Log10 LDU** | 2.4 +/- 0.1 | ND | 1.4 +/- 0.3 | ND |
|  |  |  |  |  |  |  |
| **2** | **1 day** | **LDU** | ND | 140 +/- 34 | 34 +/- 20 | 4 +/- 4 |
|  | **3 days** | **LDU** | ND | 15 +/- 11 | 3 +/- 2 | 2 +/- 2 |
|  | **1 day** | **Log10 LDU** | ND | 2.1 +/- 0.1 | 1.5 +/- 0.3 | 0.5 +/- 0.4* |
|  | **3 days** | **Log10 LDU** | ND | 1.0 +/- 0.5 | 0.4 +/- 0.3* | 0.2 +/- 0.3** |
|  |  |  |  |  |  |  |
|  |  |  | **Miltefosine tissue concentrations, mean +/- SD** | | | |
| **2** | **1 day** | **Plasma (µg/mL)** | ND | 34.8 +/- 3.6 | 40.3 +/- 2.0 | 45.6 +/- 4.1 |
|  | **1 day** | **Liver (µg /g)** | ND | 179.7 +/- 9.6 | 204.5 +/- 9.1 | 223.1 +/- 24.0 |
|  | **1 day** | **Spleen (µg /g)** | ND | 93.9 +/- 8.8 | 114.3 +/- 8.7 | 133.9 +/- 4.0 |
