# Supplementary material for: Pharmacokinetic / pharmacodynamic relationships of liposomal amphotericin B and miltefosine in experimental visceral leishmaniasis
Source: PLoS Negl Trop Dis. 2021 Mar 2;15(3):e0009013. doi: 10.1371/journal.pntd.0009013 (PMC7924795; doi:10.1371/journal.pntd.0009013)
Supplement: S3 Table — Data from experiment 2 presented here corresponds to data graphically presented in Figs 1 and 3 in the main manuscript. Data provided here in LDU was used along with the parasite burden in untreated control groups to calculate the % inhibition shown in Fig 1C. Additional data are shown for an initial dose response experiment which included a lower dose range, and for drug concentrations in the spleen compared to plasma and liver. * and ** denote that no amastigotes were detected in 2 and 3 animals of the respective groups. (DOCX) [file pntd.0009013.s003.docx]

**S3 Table**

|  | **Parasite burden, mean +/- SD, at doses of AmBisome of** | | | | | | | | |
| --- | --- | --- | --- | --- | --- | --- | --- | --- | --- |
| **Expt.** | **Read out** | **Untreated** | **0.625 mg/kg** | **1.25 mg/kg** | **2.5 mg/kg** | **5 mg/kg** | **10 mg/kg** | **20 mg/kg** | **40 mg/kg** |
| **1** | **LDU** | 655 +/- 76 | 402 +/- 55 | 407 +/- 117 | 148 +/- 35 | 100 +/- 42 | 39 +/- 23 | ND | ND |
|  | **Log10 LDU** | 2.8 +/- 0.1 | 2.6 +/- 0.1 | 2.6 +/- 0.1 | 2.2 +/- 0.1 | 2.0 +/- 0.2 | 1.5 +/- 0.2 | ND | ND |
| **2** | **LDU** | 456 +/- 65 | ND | ND | ND | 28 +/- 16 | 12 +/- 5 | 3 +/- 3* | 1 +/- 1** |
|  | **Log10 LDU** | 2.7 +/- 0.1 | ND | ND | ND | 1.4 +/- 0.3 | 1.0 +/- 0.2 | 0.4 +/- 0.4* | 0.2 +/- 0.2** |
|  |  |  |  |  |  |  |  |  |  |
|  | **Amphotericin B tissue concentrations, mean +/- SD** | | | | | | | | |
| **2** | **Plasma (µg/mL)** | ND | ND | ND | ND | 0.2 +/- 0.04 | 0.2 +/- 0.03 | 0.2 +/- 0.05 | 0.4 +/- 0.09 |
|  | **Liver (µg /g)** | ND | ND | ND | ND | 82.2 +/- 6.3 | 175.7 +/- 26.2 | 369.9 +/- 25.7 | 593.6 +/- 48.3 |
|  | **Spleen (µg /g)** | ND | ND | ND | ND | 8.0 +/- 1.6 | 19.7 +/- 1.9 | 38.3 +/- 6.1 | 146.9 +/- 60.7 |
