# Supplementary material for: Pharmacokinetic / pharmacodynamic relationships of liposomal amphotericin B and miltefosine in experimental visceral leishmaniasis
Source: PLoS Negl Trop Dis. 2021 Mar 2;15(3):e0009013. doi: 10.1371/journal.pntd.0009013 (PMC7924795; doi:10.1371/journal.pntd.0009013)
Supplement: S4 Table — Data presented here corresponds to data graphically presented in Fig 4. (DOCX) [file pntd.0009013.s004.docx]

**S4 Table**

|  |  | **Concentration, mean ± SD [µg/mL]** | **Concentration, mean ± SD [µg/g]** | |
| --- | --- | --- | --- | --- |
| **Drug** | **Time (hr)** | **Plasma** | **Liver** | **Spleen** |
| AmBisome | 0.05 | 874.9 ± 30.7 | 106.4 ± 9.6 | 51.4 ± 14.1   \| 51.4 \| 14.1 \| \| --- \| --- \| \| 80.7 \| 5.3 \| \| 83.6 \| 8.2 \| \| 96.5 \| 10.9 \| \| 103.1 \| 13.9 \| \| 113.2 \| 13.0 \| \| 96.0 \| 8.5 \|  \| 51.4 \| 14.1 \| \| --- \| --- \| \| 80.7 \| 5.3 \| \| 83.6 \| 8.2 \| \| 96.5 \| 10.9 \| \| 103.1 \| 13.9 \| \| 113.2 \| 13.0 \| \| 96.0 \| 8.5 \|   14.1 |
|  | 0.17 | 849.5 ± 38.5 | 193.8 ± 47.1 | 80.7 ± 5.3 |
|  | 0.5 | 588.9 ± 205.4 | 308.2 ± 29.9 | 83.6 ± 8.2 |
|  | 1 | 540.6 ± 39.4 | 312.9 ± 21.8 | 96.5 ± 10.9 |
|  | 3 | 381.2 ± 26.4 | 502.4 ± 69.3 | 103.1 ± 13.9 |
|  | 8 | 68.5 ± 19.4 | 730.6 ± 11.8 | 113.2 ± 13.0 |
|  | 24 | 4.1 ± 1.1 | 750.8 ± 32.2 | 96.0 ± 8.5 |
|  |  |  |  |  |
| Miltefosine | 0.24 | 0.8 ± 0.4 | 1.4 ± 0.5 | 0.4 ± 0.2   \| 0.4 \| 0.2 \| \| --- \| --- \| \| 2.2 \| 0.2 \| \| 3.8 \| 0.7 \| \| 5.7 \| 0.7 \| \| 11.9 \| 2.6 \| \| 15.0 \| 2.3 \| \| 24.5 \| 2.6 \| \| 19.1 \| 1.8 \| \| 15.4 \| 1.2 \| |
|  | 0.5 | 3.0 ± 0.4 | 5.9 ± 1.3 | 2.2 ± 0.2 |
|  | 1 | 4.0 ± 0.8 | 11.2 ± 2.7 | 3.8 ± 0.7 |
|  | 2 | 5.0 ± 0.6 | 22.8 ± 2.1 | 5.7 ± 0.7 |
|  | 4 | 7.6 ± 1.7 | 47.4 ± 10.3 | 11.9 ± 2.6 |
|  | 8 | 7.7 ± 1.1 | 55.5 ± 11.9 | 15.0 ± 2.3 |
|  | 24 | 11.8 ± 0.8 | 87.4 ± 6.4 | 24.5 ± 2.6 |
|  | 48 | 8.7 ± 0.2 | 60.7 ± 6.0 | 19.1 ± 1.8 |
|  | 72 | 7.0 ± 0.6 | 51.8 ± 6.2 | 15.4 ± 1.2 |
